# A Parametric Study of Optimum Road Modular Hinged Frames by Hybrid Metaheuristics

**DOI:** 10.3390/ma16030931

**Published:** 2023-01-18

**Authors:** Andrés Ruiz-Vélez, Julián Alcalá, Víctor Yepes

**Affiliations:** Institute of Concrete Science and Technology (ICITECH), Universitat Politècnica de València, 46022 València, Spain

**Keywords:** reinforced concrete, modular, hinged frame, hybrid metaheuristic, parametric, regression

## Abstract

This paper addresses a study of cost-optimal road modular hinged frames. The performance of three hybrid metaheuristics is assessed through a fractional factorial design of experiments. The results allow for selecting and calibrating the hybrid simulated annealing to solve the combinatorial optimization problem. By varying the horizontal span from 8 to 16 meters and the earth cover from 1 to 5 meters, 25 different structural configurations are studied. The calibrated methodology is applied to obtain nine different frames with optimal costs for each configuration. The study of the economic, environmental and geometrical characteristics of the 225 optimum structures allows for the development of a regression analysis. With $R^{2}$ correlation coefficients close to the unit, the expressions form a valuable tool for calculating the final cost, associated emissions, embodied energy and particular geometric characteristics. The optimum structures present slender and densely reinforced designs. In addition, some structures show considerable reductions in the shear reinforcement, something solved by localized increases in longitudinal reinforcement.

## 1. Introduction

There is a growing general interest in caring for the environment. Widespread awareness of the consequences of pollution promotes sustainable alternatives over traditional ones [1,2,3]. In this context, construction is one of the most contributing industries to global pollution. Currently, in addition to fulfilling their service, infrastructures must incur the lowest possible environmental impact [4,5,6].

However, improving the structural design process is a challenging task [7,8,9]. A large number of discrete variables must be considered. Therefore, systematizing the obtaining of designs is very complicated. This complexity leads to the traditional design process depending on the responsible technician [10]. Based on existing structures, the structural engineer defines an initial design. It is then checked, and the grade or quantity of materials is modified if it does not meet structural requirements. This process is repeated, achieving a safe structure that, on the other hand, does not make optimal use of the materials. One way of improvement consists of approaching the design process of reinforced concrete structure as a combinatorial optimization problem [11,12,13,14,15]. Its solution results in obtaining non-traditional designs that, in addition to verifying the structural requirements, make optimum use of materials.

The techniques applied to solve combinatorial optimization problems can be divided into two main groups: exact techniques and approximate or metaheuristic techniques. The former achieve the best possible result and are suitable for problems with a limited number of variables [16,17]. The latter allow for the study of more complex problems, obtaining high-quality results. This characteristic generates a general interest in applying approximate techniques to solve structural problem designs [7,18,19,20].

These approximate methods make use of a variety of metaheuristic algorithms based on different working principles. This allows the conditioned exploration of the solution space with a certain degree of randomness. The techniques considered in the present study can be framed within the so-called hybrid metaheuristics. Based on the simulated annealing (SA), threshold accepting (TA) and old bachelor’s acceptance (OBA) algorithms, these integrate a mutation operator (MO) specific to genetic algorithms (GA). The SA, TA and OBA algorithms classify as local search algorithms. Starting with a feasible solution, these obtain and evaluate new solutions similar to the current one through a series of rules. In contrast to these, GAs search for optimal solutions by selection, crossover and mutation of populations of feasible solutions. Hybridizing both typologies allows for high-quality results in optimizing structures such as composite bridges [20,21], retaining walls [15,22,23,24] or prestressed box girder bridges [21,25].

In this context, the present work develops a parametric study of road modular hinged frames (RMHF). Reinforced concrete frames are a very common structural typology in transport infrastructure. With spans ranging between three and twenty meters, these structures are applicable as a solution for road crossings. Previous work focused on studying cast-in-place road frames (CPRF) [26]. However, the analysis of the related literature allows for identifying a lack of development in the study of RMHF. Based on the quality and consistent characteristics of prefabrication, the present study solves the lack of current development and improves the general knowledge about optimal RMHF. In this way, the precast typology, considered an alternative to the cast-in-place structures, is proposed.

**Figure 1:**
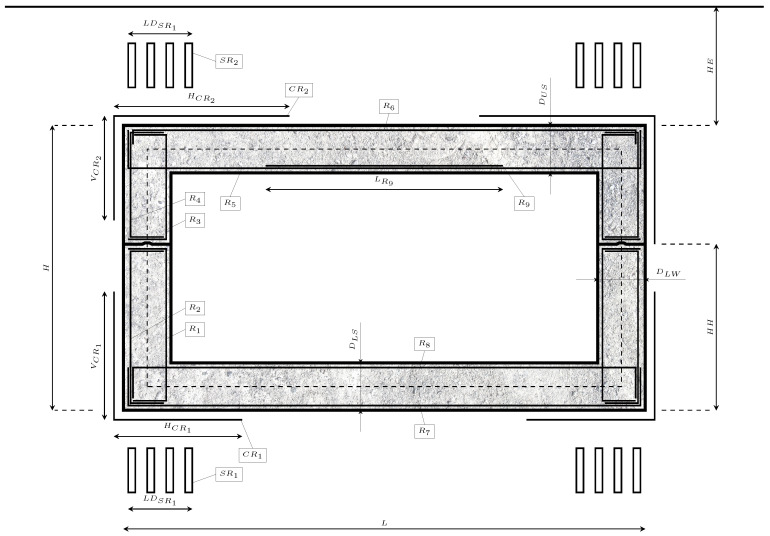
Set of variables of the RMHF considered in the optimization problem.

The RMHF comprises two sets, which facilitate transport to the construction site. The two sets present three main structural components, the upper and lower slabs and the lateral walls. Traditionally, the slab’s thickness is established between 1/15 and 1/10 of the span. The lateral walls’ thickness ranges between 1/12 of the vertical span and the particular slab thickness. Four main geometric characteristics define the RMHFs: the clear height (*H*), the horizontal span (*L*), the height of the hinge (HH) and the earth cover above the structure (HE). Figure 1 represents the detailed RMHF desgin.

The study considers spans ranging between 8 and 16 m and earth cover depths between 1 and 5 m. The study of the optimal designs allows for determining the influence of the characteristics that define them and developing a regression analysis. In addition, the comparison with the cast-in-place alternative allows for establishing a series of improvements derived from the prefabrication of this structural typology.

This way, the study develops a factorial design of experiments (DoE) in order to evaluate the performance of three hybrid metaheuristics: simulated annealing with mutation operator (SAMO), threshold accepting with mutation operator (TAMO) and old bachelor’s acceptance with mutation operator (OBAMO). Analysing the results allows for calibrating the parameters that define the algorithms and the subsequent selection of the best-performing technique [27]. Once selected, the hybrid metaheuristic is applied to obtain optimal RMHF. Obtaining optimal designs requires the establishment of an objective function. The economic cost of a structure is a direct representation of the use of materials. Thus, the final cost of RMHFs is optimized for 25 different horizontal span and earth cover combinations. Nine designs are optimized for each configuration, obtaining a total of 225 environmentally efficient designs under highly restrictive budgets.

## 2. Optimization Problem

This section details the combinatorial optimization problem proposed. This comprehends the complete definition of the variables, parameters, constraints and objective function [28]. The problem posed consists of minimizing the use of materials in the construction of RMHFs. The structure’s final cost $C(\vec{x})$, calculated by means of Equation (1), was considered the objective function. Its calculation is direct, consisting of the multiplication of the unit price of each material $c_{i}$ and the quantity used $m_{i}\left(\vec{x}\right)$. In addition, to analyse the optimal designs’ environmental impact, the associated CO_2_ emissions $AE(\vec{x})$ and embodied energy $EE(\vec{x})$ were evaluated through Equations (2) and (3). Similar to the objective function, emissions are obtained as a factor of each material’s quantity $m_{i}\left(\vec{x}\right)$, the unitary emissions $ae_{i}$ and unitary embodied energy $ee_{i}$, respectively. The designs must comply with a series of constraints $R(\vec{x})$ detailed in Section 2.3. The compliance of the optimal designs with the constraints is generally expressed through Equation (4).

(1) $$\begin{array}{c} \begin{array}{c} C\left(\vec{x}\right)=\sum_{i=1}^{n}c_{i}\cdot m_{i}\left(\vec{x}\right) \end{array} \end{array}$$

(2) $$\begin{array}{c} \begin{array}{c} AE\left(\vec{x}\right)=\sum_{i=1}^{n}ae_{i}\cdot m_{i}\left(\vec{x}\right) \end{array} \end{array}$$

(3) $$\begin{array}{c} \begin{array}{c} EE\left(\vec{x}\right)=\sum_{i=1}^{n}ee_{i}\cdot m_{i}\left(\vec{x}\right) \end{array} \end{array}$$

(4) $$\begin{array}{c} \begin{array}{c} R(\vec{x})\leq 0 \end{array} \end{array}$$

The set of unit cost $c_{i}$, unitary associated CO_2_ emissions $ae_{i}$ and embodied energy $ee_{i}$ for each of the materials can be consulted in Table 1. These values were obtained from the Construction Technology Institute of Catalonia by the BEDEC database [29].

### 2.1. Variables

A total of 38 variables defined each RMHF design solution, establishing the geometry, grade of materials and passive reinforcement design. The value of the variables represented in Figure 1 was subjected to optimization within the ranges presented in Table 2. Meanwhile, parameters were established previously and remained constant during the optimization process. The influence of the main geometrical parameters was the subject of a parametric study shown in Section 4.

Prior to solving the problem, all variables were discretized. This way, the variables could take a given number of values within the range stipulated for each. The geometric variables adopted thicknesses every two centimetres within limits. The separation between shear reinforcement branches was established every five centimetres within the stipulated range. Normalized reinforcement diameters between 10 and 35 millimetres were considered. Moreover, the number of bars was set to any integer lower or equal to the maximum value established.

Due to the number of variables and values each was allowed to take, the problem’s solution space presents a dimension of $9.87\cdot 10^{43}$. This large magnitude states that a complete study of the solution space is not feasible. Thus, the application of the techniques described in Section 3 is particularly interesting. The calibration and selection of the hybrid metaheuristic allow for obtaining high-quality results while maintaining adequate computational costs.

### 2.2. Parameters

There are several determining factors in the design of RMHF. The first of these is the vertical span (*H*). Based on common characteristics of this typology, a vertical span of five meters was established. The second factor is the height of the hinge (HH), whose position relative to the lower slab was set at 3/5 of the vertical span. The remaining two parameters were the subject of the parametric study. The variation of their magnitude gave rise to optimum designs whose study allowed a regression analysis. The first one is related to the horizontal span (*L*). RMHFs functioning as a solution for road crossings bridge spans ranging from 8 to 16 m. Therefore, RMHFs were optimized in which *L* adopted 8, 10, 12, 14 and 16 m. This type of buried structure is usually located in up to 12 m deep embankments. Considering the established *H*, the study adopted earth cover depths of 1, 2, 3, 4 and 5 m for a medium quality soil. The set of parameters and all those necessary for the calculation and verification of the RMHF can be consulted inTable 3.

Loads associated with heavy vehicles and Marston’s effect were considered according to the Spanish regulation recommendations [32,33]. The remaining loads, actions and associated conditions were taken from current applicable regulations [30,31]. As a completely buried structure, temperature variations occur slowly and homogeneously throughout the surrounding earth’s mass. Following the current standard’s recommendations [30,33], it is feasible to consider that no loads are derived from temperature gradients. Thus, the thermal effects were disregarded. In addition, since prefabricated structures undergo intensive control in the manufacturing and curing process, the rheological effects were neglected. Finally, a generic location was established within a non-seismic zone, so consideration of the associated actions was not necessary.

### 2.3. Constrains

As stated in Section 2, the optimal RMHF design is subject to several constraints. These ensured adequate representation of the reality and the resulting structure meeting the structural requirements of current regulations [30,31]. The constraints applied are divided into Ultimate Limit States (ULS) and Service Limit States (SLS). ULS ensure structural integrity, while SLS ensure adequate service life. Prior to verification, it is necessary to calculate the structure’s internal stresses. This was achieved by applying the displacement method to the two-dimensional model under the linear elastic analysis hypothesis.

Once the internal stresses were known, the shear ULS was checked. This consisted of comparing the acting shear stress with the standard’s exhaustion limit values stipulated. In addition, the stress increase in the tensile reinforcement associated with the shear stress was calculated. If the structure complied with the shear ULS, the tensile increment was applied, and the normal stresses ULS were checked. This consisted of verifying that the axial stress and bending moment are located within the interaction diagram of each section. After this, the deflection ULS was checked, limiting the displacement of the upper slab to 1/250 times the frame span. Subsequently, compliance with the fatigue ULS was ensured.

Within the second group of constraints, the cracking SLS was checked. In order to do so, the crack opening was obtained and compared with the standard’s limit value. In addition, the maximum and minimum passive reinforcement and crack control reinforcement amounts were also checked. Constructibility was reviewed by calculating the spacing between bars. Finally, the transversal reinforcement was obtained directly from the flexural passive reinforcement design.

The developed software allowed verifying the ULS and SLS according to the described process. The result of the verification process was a series of coefficients generally expressed by Equation (5). Associated with the ULS and SLS verification, these resulted from the relation between each section’s acting ($A_{s}$) and resistant ($R_{s}$) stresses.

(5) $$\begin{array}{c} \begin{array}{c} \frac{A_{s}}{R_{s}}\geq 1 \end{array} \end{array}$$

## 3. Proposed Hybrid Metaheuristic Strategies

This section details each of the hybrid metaheuristics considered in the study. A DoE, detailed in Section 3.4 was carried out to calibrate and select the best-performing technique. Once selected, it was applied to obtain the optimal design analysed in the parametric study presented in Section 4. Initially, the SAMO, TAMO and OBAMO hybrid metaheuristics were considered. The following sections detail the functioning of each.

### 3.1. Hybrid Simulated Annealing

The SAMO algorithm was the first of the three techniques considered. The basis of the technique is the SA, proposed by Kirkpatrick et al. [34] for electrical circuit design. The SAMO’s novelty consists of adding a mutation operator to the SA. For specific problems, this hybridization results in considerable improvements in the exploration capability of the algorithm [7,16,23]. The algorithm bases its operation on the thermal annealing process. The annealing is applied to certain materials to modify their mechanical characteristics. These changes are achieved thanks to the microstructural modifications during the thermal annealing. The temperature is the controlling parameter of the process. Initially, it has a high value, which is gradually reduced by applying the cooling coefficient. During this period, the material adopts different crystallization states with lowering energy levels. The SA equates each feasible solution to the problem with a physical crystallization state. Consequently, the objective function is equivalent to the internal energy associated with each state.

The SAMO generates and evaluates the cost of an initial random solution. Then generates a new solution by applying a movement, which is affected by the mutation operator. The newly generated solution is evaluated and directly accepted if it costs less. If the new cost worsens, it is accepted if the probability *P* obtained through Boltzmann energetic expression, Equation (6), is greater than a random value in the 0 to 1 range. The acceptance probability *P* depends on the difference between the current and new objective function values ΔE and the current temperature *T* [34]. This process is repeated at the same temperature for a predetermined number of iterations called the Markov chain. When a chain is finished, the temperature decreases geometrically at the rate of the cooling coefficient. The temperature reduction progressively limits the SAMO’s ability to accept solutions worse than the current one.

(6) $$\begin{array}{c} \begin{array}{c} P=e^{-(\Delta E/T)} \end{array} \end{array}$$

The optimization process ends when one of two criteria is met. The stop criterion establishes the maximum number of chains in which a bettering solution is not found. On the other hand, the termination criterion dictates a minimum percentage of the initial temperature of the problem. The initial temperature setting is carried out as proposed in Medina [35]. Correctly calibrating this parameter is essential in order to obtain high-quality results [25,36].

Five parameters define the SAMO, three of which are specific to the SA algorithm. The Markov chain length (MCL), the cooling coefficient (CC) and the stopping criterion (SC). The remaining two determine the standard deviation (SD) and the number of variables (VN) affected by the mutation operator. The values considered for the DoE are detailed in Section 3.4.

### 3.2. Hybrid Threshold Accepting

The second alternative assessed in the study was the TAMO. This technique is based on the TA, which Dueck and Scheuer [37] developed as an improvement for the SA. The TA and the SA differ in the acceptance criterion for worsening solutions. As described in Section 3.1, the SA has a probabilistic criterion. On the other hand, the TA accepts worse solutions by directly comparing the cost difference and a threshold.

The initial threshold is established analogously to the SA [35]. It is then geometrically reduced at the rate of the reduction coefficient. Limiting the ability to accept worse solutions as the optimization develops. Like the SAMO, five parameters define the TAMO. The chain length (CL), the reduction coefficient (RC) and the stopping criterion (SC) are specific to the TA. The remaining two are the mutation operator’s standard deviation (SD) and the number of affected variables (VN). Similar to the SAMO, the process finishes when either the SC or the termination criterion are met.

### 3.3. Hybrid Old Bachelor’s Acceptance

The third technique studied was the OBAMO. Consisting of a modification of the OBA proposed by Hu et al. [38], it works similarly to the TAMO. The main difference is that the threshold does not start from a high initial value and then decreases over time. In the OBAMO, the initial threshold is zero. Then, it increases or decreases depending on the rejection or acceptance of new solutions. The criterion of acceptance of worsening solutions is deterministic. As in the TAMO, worsening solutions are accepted if the cost increase is lower than the threshold at that time.

In this context, the OBAMO is also defined by five parameters. Two establish the standard deviation (SD) and the number of variables (VN) affected by the mutation operator. Two of the remaining are related to the threshold increase (IC) and decrease (DC) coefficients. These are applied each time a solution is rejected or accepted, respectively. Unlike the algorithms described in Section 3.1 and Section 3.2, the OBAMO does not have a control parameter that decreases in time. Therefore, the fifth parameter is the termination criterion (TC), equivalent to the algorithm’s maximum number of iterations.

### 3.4. Design of Experiments

The hybrid metaheuristic parameters’ calibration directly impacts the optimization results’ quality. Each technique’s performance varies depending on the studied optimization problem. Thus, selecting the best-suited algorithm for the considered problem is crucial. In addition, it is essential to appropriately calibre the metaheuristic parameters. In this context, the DoE detailed in this section allowed the selection and application of the technique with the best performance.

The study developed a $2^{\left(n-1\right)}$ fractional factorial DoE for each hybrid metaheuristics. Two levels were established for each parameter, corresponding with the values presented in Table 4. A total of parameters define the three techniques, so 16 combinations of each parameter’s level need to be considered. In order for the results to be statistically representative, each of the configurations is run five times. Therefore, the influence of each of the parameters of the hybrid metaheuristics can be addressed. Table 5 presents the mean results of the DoE for each configuration.

**Figure 2:**
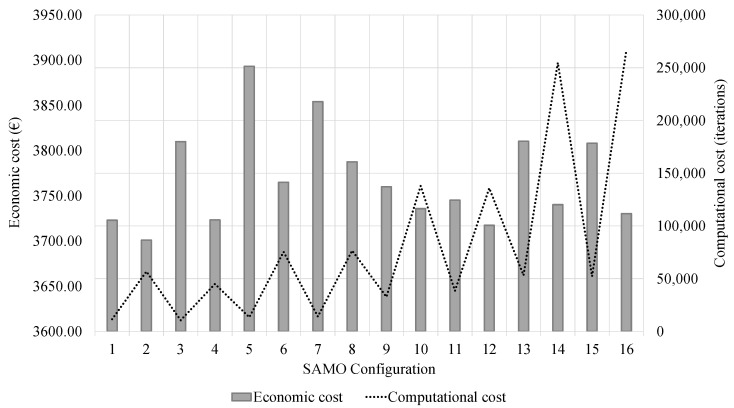
DoE results for the SAMO hybrid metaheuristic.

The algorithm’s performance does not depend solely on the final result quality. Thus, in addition to analysing the lowest costs, the computational cost associated with obtaining each optimal RMHF was addressed. The analysis of the DoE results allows for identifying the SAMO as the best performing technique for its application to solving the problem posed. Furthermore, considering the associated computational cost, the algorithm was calibrated so that the MCL was 10,000 iterations, with CC of 0.8 and SC of 5 chains without improvement. In addition, the mutation operator was defined by an SD of 0.1 and affected a VN of 5. In general, as Figure 2 shows, longer chain lengths achieve better results.

However, the computational cost increases rapidly. In this context, the calibration achieved an optimum RMHF whose cost differed only 0.006% from the general optimum. However, the computational cost is 4.95 times lower. Moreover, with a means standard deviation of 0.597%, the algorithm is a robust tool for solving the problem. The algorithm was applied in order to obtain optimum RMHF analysed in the parametric study detailed in Section 4.

## 4. Results of the Parametric Study and Regression Analysis

The present work carried out the parametric study of optimal RMHF obtained by applying the SAMO hybrid metaheuristic described in Section 3 to the combinatorial optimization problem described in Section 2. Five horizontal spans of 8, 10, 12, 14 and 16 m were considered. In addition, five earth cover depths of 1, 2, 3, 4 and 5 m were considered. The combination of both parameters allowed the study of 25 different RMHF configurations. The calibrated SAMO was applied, and nine optimal structures were obtained for each configuration. The analysis of a total of 225 optimal RMHF allowed the study of the main economic, geometrical and design characteristics.

Furthermore, the regression analysis was carried out, obtaining good fitting results. The expressions obtained allow for structural techniques to calculate certain approximations prior to RMHF design. This section focuses on the presentation and discussion of the results obtained.

### 4.1. Final Cost Analysis

**Figure 3:**
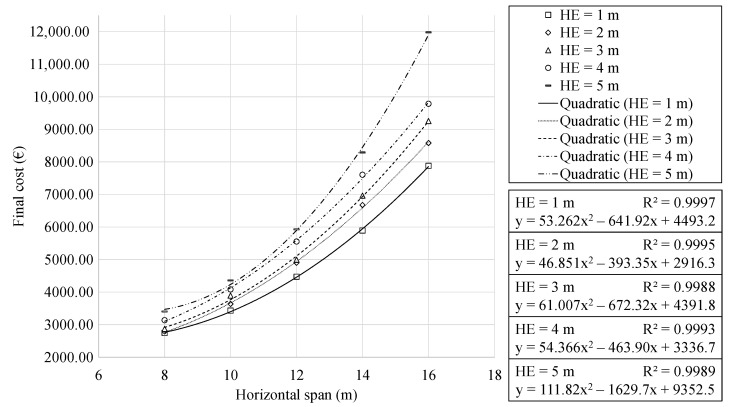
Cost of the RMHF as function of the horizontal span, for each earth cover depth.

Figure 3 represents the mean minimum final cost of the optimum RMHF as a function of the span. The results showed a clear quadratic relation in all cases. Increasing the size of the RMHF causes the loads associated with self-weight and soil fill to increase linearly. Consequently, the shear and bending moments vary linearly and quadratically, respectively. In addition, deflections vary at a two-quadratic rate. Together, these variations make it necessary to increase the amount of resistant material in the RMHF.

The final cost of the structure is a direct representation of the use of materials. The results allowed associating the variation in the final cost to two reasons. The first is the intrinsic need to use more material to build a larger structure. The second is that the structure needs to resist stresses of greater magnitude. The first reason justified a linear increase in the final cost. The study of the second allowed establishing the increase in shear and bending moment stresses as the direct cause behind the quadratic relation identified in the regression analysis. With an $R^{2}$ regression coefficient between 0.9988 and 0.9997, the expressions obtained are an adequate tool for the final cost approximation of the RMHF. It is relevant to highlight that when the earth cover above the structure is five meters, the final cost increases 1.44 times when the span increases from 14 to 16 m. This factor is, to some extent, more significant than other earth cover cases, varying between 1.28 and 1.33 for 1 and 4 m, respectively.

**Figure 4:**
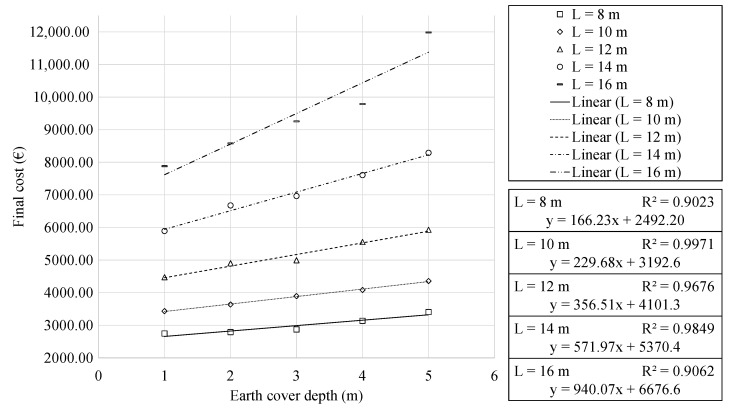
Cost of the RMHF as function of the earth cover, for each horizontal span.

Figure 4 shows the mean minimum cost as a function of earth cover depth. The regression analysis showed a linear relation. With slightly lower correlation coefficients, the expressions obtained represent the final cost as a function of the earth cover depth. The analysis of the results allowed for associating the linear relation to the linear increase or decrease in the axial forces of the RMHF when varying the earth cover. Increasing the earth cover by one meter results in final cost increases between 5.73% and 10.49% for the 8 and 16 m frames, respectively. This corresponds to an increasing slope of the regression trendlines as the earth cover increases.

Furthermore, the analysis of the results allowed identifying that increments in the larger span range relate to more significant increases in the final cost. In Figure 3, this is represented by faster growth of the cost curve. Whereas in Figure 4, it corresponds to an increasing separation between the straight trend lines.

### 4.2. Sustainability Analysis

This section focuses on the results related to the associated CO_2_ emissions and embodied energy of the optimum RMHF. The regression analysis allowed identifying similar characteristics to those mentioned for the final cost in section ref. In both cases, there is a quadratic relationship with the span. In addition to a linear relation to the earth cover depth.

**Figure 5:**
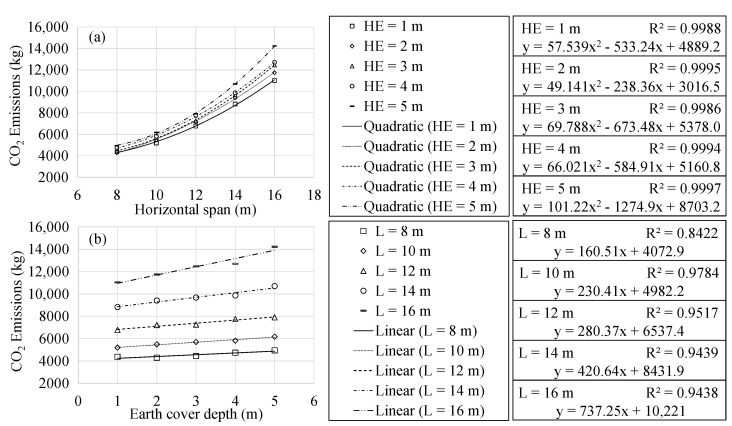
Associated CO_2_ emissions analysis as a function of: (**a**) horizontal span; (**b**) earth cover.

**Figure 6:**
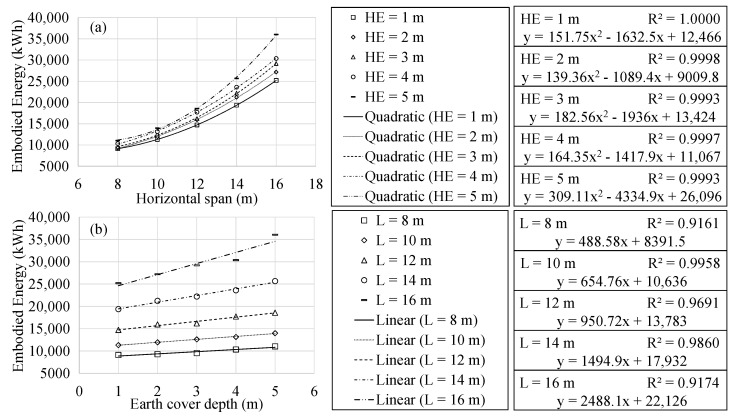
Embodied energy analysis as a function of: (**a**) horizontal span; (**b**) earth cover.

The present work considered associated CO_2_ emissions and embodied energy as impact measuring tools associated with the optimal RMHF obtained. However, these were not considered objective functions. Thus, the particularized study of the characteristics of optimal frames as a function of such variables is beyond the scope of the study. Figure 5 and Figure 6 show the results obtained in the regression analysis. With $R^{2}$ correlation coefficients close to one, the expressions form rough impact measuring tools for the design of RMHFs.

### 4.3. Geometrical Characteristics Analysis

**Figure 7:**
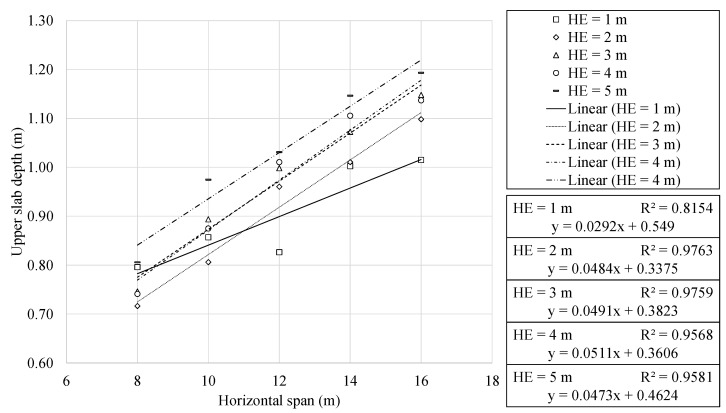
Upper slab depth as a function of the horizontal span.

**Figure 8:**
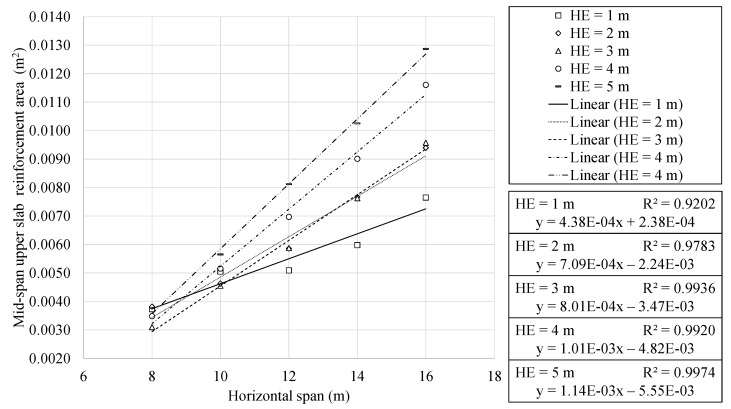
Upper slab mid span reinforcement area as a function of horizontal span.

This section continues with the results of the study. In this context, the relevant geometric characteristics of the optimal RMHFs were analysed. Figure 7 and Figure 8 show the top slab depth and mid-span reinforcement area as a function of the horizontal span. The regression analysis showed a clear linear relationship between the upper slab depth and horizontal span. The results present a similar trend to those obtained in previous studies of similar structures [11,26]. The optimization problem posed does not condition the design of passive reinforcement with traditional considerations. The individualized study of specific variables resulted in expressions with correlation coefficients somewhat lower but still representative. This is somewhat to be expected when studying particular variables. Unlike the final cost or sustainability indicators, the precise analysis of a single variable makes the consequences of the discretization process relatively more noticeable.

For an earth cover of one meter, the upper slab depth and horizontal span ratio vary between 10.05 and 9.92 for horizontal spans of 8 and 16 m, respectively. For structures buried at five meters deep, this ratio varies between 15.76 and 15.40 for the same cases. The expressions result of the regression analysis allowed for calculating a mean variation factor of 1.47 as the span increased from 8 to 16 m.

The reinforcement area in the mid-span section of the upper slab showed a clear linear relationship with the horizontal span of the RMHF. The passive reinforcement area increases with an average factor of 2.82 as the span varies from 8 to 16 m.

It is of particular interest to note that the RMHF configuration buried at one meter presents a behaviour that differs from the one presented by the rest of the depths. This characteristic behaviour is seen when studying Figure 7 and Figure 8. In both figures, it can be seen that the slope of the line corresponding to 1 m of burial depth is less pronounced than the others. An in-depth analysis of the designs corresponding to this configuration allows us to identify the frames with 8 and 10 m of horizontal span responsible for this behaviour. These configurations showed upper slabs with greater depths and higher flexural reinforcement. Such characteristics result from RMHF designs whose shear reinforcement showed considerable reductions. The problem posed made use of combinatorial optimization to obtain the designs. Thus, there is no predisposition for the traditional design. Therefore, the reduction in shear reinforcement in the one-meter deep, 8 and 10 m horizontal span RMHF was solved with localized increases of flexural reinforcement and greater depth sections.

Generally, the designs presented lower slab average depths values of 57.18% of the upper slab depth. Therefore, on average, the upper slab depth is 1.75 greater than the lower slab. In the case of the lateral walls, the optimum RMHFs presented reduced depths. On average, the lateral wall depth equalled 47% of that of the upper slab. The identified ratios provide an initial approximation in the design of the structural typology. However, the proposed reinforcement design depends on a substantial set of variables. Thus, the scope of the study makes a particularized analysis of each of them unfeasible. The results presented are a functional tool to be considered by structural technicians with pertinent technical backgrounds.

### 4.4. Materials Analysis

**Figure 9:**
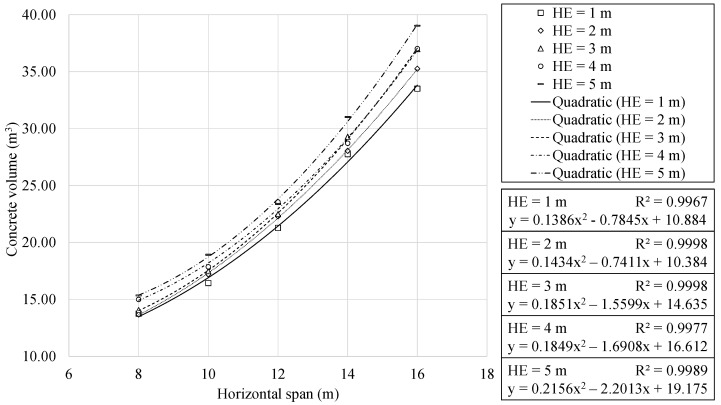
Concrete volume as function of horizontal span, for each earth cover depth.

**Figure 10:**
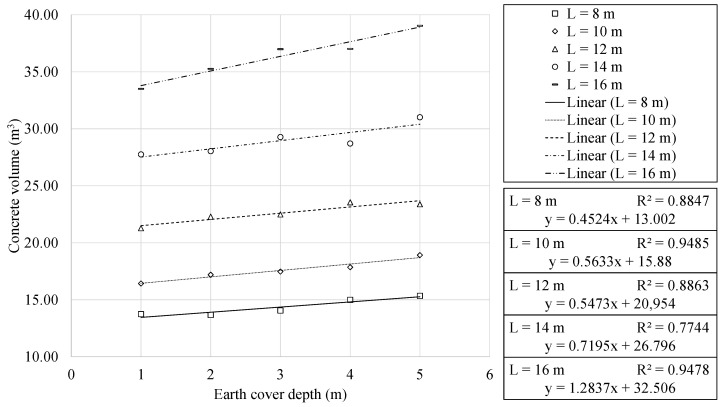
Concrete volume as function of earth cover depth, for each horizontal span.

Section 4.3 highlighted the main geometric characteristics that were identified. This section focuses on the analysis of the materials used in the building of the optimum RMHFs. Figure 9 and Figure 10 show the volume of concrete required as a function of the span and the earth cover depth. Concrete usage showed a quadratic relationship with the horizontal span. Meanwhile, the analysis of the results indicated a linear relationship with the earth cover depth. The regression analysis allowed for obtaining expressions with $R^{2}$ correlation coefficients very close to unity. In all cases, the expressions where concrete volume is expressed as a function of the horizontal span perform an approximation of greater quality when compared to those where the concrete volume is a function of the earth cover.

A total of 212 out of the 225 optimal frames studied make use of concrete grade C25/30. The use of higher grade concrete leads to more slender designs. However, the fact that 94.22% of the optimal RMHF designs used the lowest grade within the possible options shows that using a higher grade is not the most economical solution. In this context, the expressions obtained in the regression analysis are a useful tool for calculating the C25/30 grade concrete usage. Based on the results presented in Table 1, it was observed that the increase of 1 m in the earth cover leads to an increase of EUR 40.20 and 114.07 per linear meter in the 8 and 16 m span frames, respectively. Furthermore, as a consequence, 182.06 and 516.61 additional kWh are required for the aforementioned spans. This leads to an additional 116.11 and 329.47 kg of CO_2_ emitted into the atmosphere.

**Figure 11:**
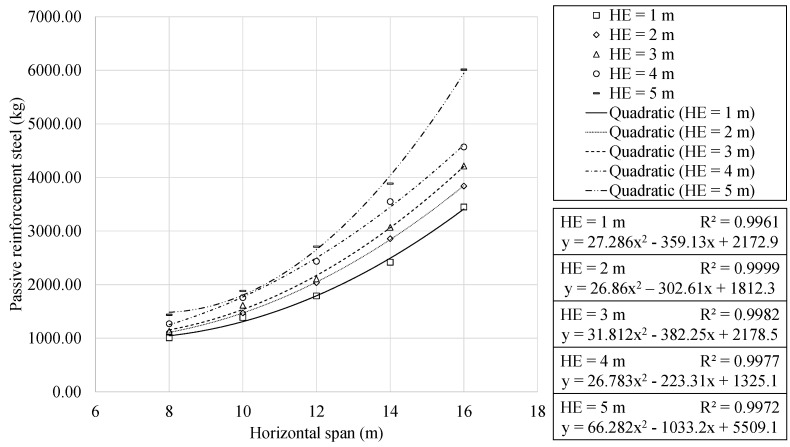
Passive reinforcement steel as function of horizontal span, for each earth cover depth.

**Figure 12:**
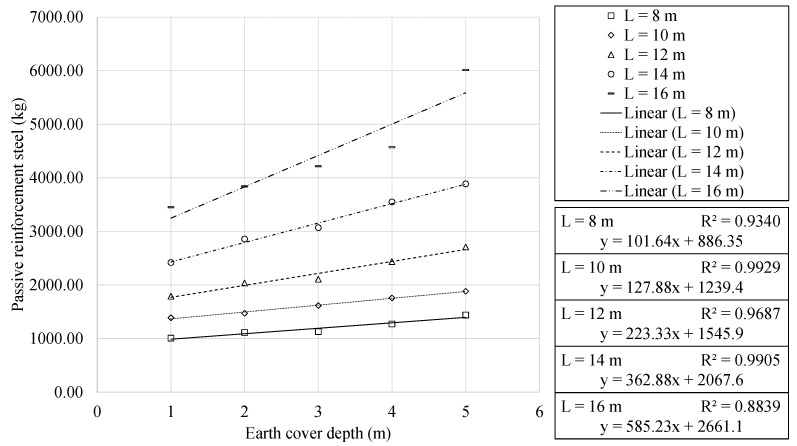
Passive reinforcement steel as function of earth cover depth, for each horizontal span.

The analysis of the amount of steel used for passive reinforcement is presented in Figure 11 and Figure 12.

Similar to the behaviour highlighted for concrete volume, quadratic and linear relationships are observed with the span and earth cover, respectively. Furthermore, the results showed a clear trend towards using a specific steel grade. This led to 91.11% of the optimal RMHFs using B500S steel for the passive reinforcement. The regression analysis obtained expressions with R2 correlation coefficients close to one. A one meter increase in the earth cover leads to an increase in steel use of 101.64 and 585.23 kg for the 8 and 16 m span RMHFs, respectively. As stated in Section 1, the final cost of the structure is a direct representation of the use of materials. Thus, the higher usage of steel mentioned above, leads to an increase in the final cost of EUR 144.32 and 831.02 per linear meter, respectively. Similar to that mentioned for concrete use, the increase in steel necessary is also related to higher energy consumption and CO_2_ emissions.

**Figure 13:**
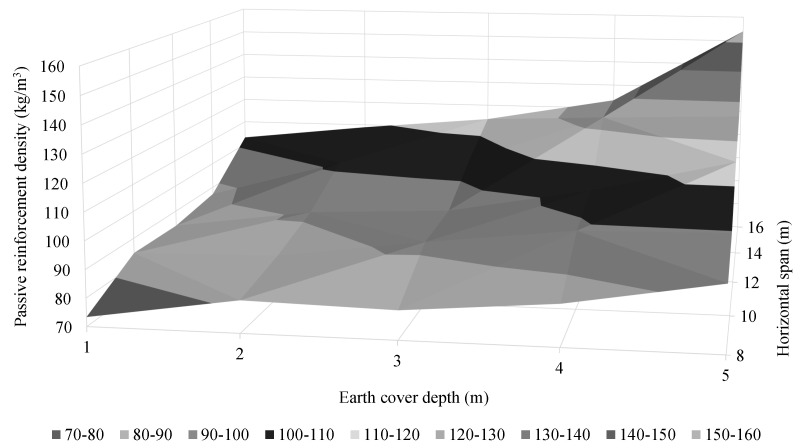
Reinforcement density distribution surface for the optimum RMHFs configurations studied.

The passive reinforcement design of the RMHFs studied depends on a large number of variables. The scope of the study allows for considering the most relevant characteristics. Thus, Section 4.3 described results relating to the passive reinforcement area in the mid-section of the upper slab. In addition, another relevant result regarding the passive reinforcement design is the overall reinforcement density of the structure. In this context, Figure 13 shows the passive reinforcement density distribution surface as a function of the horizontal span and earth cover depth.

The optimum RMHFs presented quite dense passive reinforcement designs when compared with similar structural solutions [10,11,26]. With densities ranging from 73.34 to 153.99 kg/m${}^{3}$. The increase from 8 to 16 m span led to an average increase of 1.45 in the passive reinforcement density. In addition, the study identified the influence of the earth’s cover depth. Similarly to the case above, the increase in earth cover depth factors the reinforcement density by 1.35.

## 5. Conclusions

The present work examined the parametric study of optimal RMHFs. For this purpose, the study approached the structure’s design as the combinatorial optimization problem described in Section 2. The use of three hybrid metaheuristic algorithms was considered for solving the problem. A fractional factorial DoE was carried out, the results of which placed the SAMO as the most suitable technique. In addition, the analysis of the DoE results allowed calibration of its parameters to obtain the best possible performance. The metaheuristic techniques and the DoE were detailed in Section 3. Considering spans between 8 and 16 m and earth cover depths from 1 to 5 m, the study analysed 25 different RMHF configurations. The SAMO was applied to obtain nine optimal RMHFs for each configuration. The economic optimization allowed the analysis of 225 RMHF designs. By studying the main characteristics, a regression analysis was developed. The analysis obtained representative expressions with $R^{2}$ regression coefficients close to one. In view of the results presented in Section 4, the authors consider it appropriate to note the following conclusions:

- The hybridization of local search-based algorithms with GA mutation operators gives rise to hybrid metaheuristics. These techniques are applicable in automating the optimal design of precast structures. The SAMO presents the best performance in solving the problem posed. The calibrated method has Markov chain lengths of 10,000 iterations, a cooling coefficient of 0.8 and a stopping criterion of 5 chains without improvement. In addition, the mutation operator affects one variable with a standard deviation of 0.1.
- The cost and environmental impact meters present an excellent fitting quadratic relationship when studied as a function of the horizontal span. This relationship is linear when considered a function of the earth cover depth. The expressions obtained are representative and form a valuable tool for the approximate calculation of the final cost, the associated CO_2_ emissions and the embodied energy of RMHFs.
- The RMHF design depends on a large number of variables. The study of each particular variable lands out of the scope of the present work. However, optimal structures present reduced depths with dense reinforcement designs. This density increases with both span and earth cover depth. In addition, the mid-span upper slab reinforcement area shows quadratic and linear relationships with the span and burial depth, respectively.
- Previous designs do not condition the structures conceived using the proposed methodology. Thus, any configuration that verifies the requirements is considered a feasible solution. In this context, the 8 and 10 m span RMHF buried one meter deep presented specific characteristics that differ from the general. With considerable reductions in shear reinforcement, these structures have upper slabs with greater depth and mid-span reinforcement areas.

## Figures and Tables

**Table 1:** Unit cost, associated CO_2_ emission and embodied energy values for each material [29].

Unit	Material	Unit Cost (EUR)	CO_2_ Emissions (kg)	Energy (kWh)
m${}^{3}$	C25/30 Concrete	88.86	256.66	402.44
m${}^{3}$	C30/37 Concrete	97.80	277.72	428.29
m${}^{3}$	C35/45 Concrete	101.83	278.04	429.95
m${}^{3}$	C40/50 Concrete	104.83	278.04	429.95
kg	B 400 S	1.40	0.70	3.38
kg	B 500 S	1.42	0.70	3.38

**Table 2:** Variables considered in the optimization problem.

Geometrical Variables			Num. Values	Range Values
Upper slab depth	(m)	$D_{US}$	46	0.30 to 1.20
Lower slab depth	(m)	$D_{LS}$	41	0.40 to 1.20
Lateral walls depth	(m)	$D_{LW}$	46	0.30 to 1.20
**Materials variables**				
Concrete grade	(MPa)	*C*	4	25, 30, 35 or 40
Steel grade	(MPa)	*S*	2	400 or 500
**Passive reinforcement variables**				
Flexural reinforcement $R_{1}$	(mm)	$\phi_{R_{1}}$	6	10 to 32
	(bars)	$n_{R_{1}}$	9	4 to 12
Flexural reinforcement $R_{2}$	(mm)	$\phi_{R_{2}}$	6	10 to 32
	(bars)	$n_{R_{2}}$	9	4 to 12
Flexural reinforcement $R_{3}$	(mm)	$\phi_{R_{3}}$	6	10 to 32
	(bars)	$n_{R_{3}}$	9	4 to 12
Flexural reinforcement $R_{4}$	(mm)	$\phi_{R_{4}}$	6	10 to 32
	(bars)	$n_{R_{4}}$	9	4 to 12
Flexural reinforcement $R_{5}$	(mm)	$\phi_{R_{5}}$	6	10 to 32
	(bars)	$n_{R_{5}}$	9	4 to 12
Flexural reinforcement $R_{6}$	(mm)	$\phi_{R_{6}}$	6	10 to 32
	(bars)	$n_{R_{6}}$	10	3 to 12
Flexural reinforcement $R_{7}$	(mm)	$\phi_{R_{7}}$	6	10 to 32
	(bars)	$n_{R_{7}}$	9	4 to 12
Flexural reinforcement $R_{8}$	(mm)	$\phi_{R_{8}}$	6	10 to 32
	(bars)	$n_{R_{8}}$	9	4 to 12
Flexural reinforcement $R_{9}$	(mm)	$\phi_{R_{9}}$	6	10 to 32
	(bars)	$n_{R_{9}}$	9	4 to 12
	(m)	$L_{R_{9}}$	151 to 451	3 to 0.75 ·L
Corner reinforcement $CR_{1}$	(mm)	$\phi_{CR_{1}}$	6	10 to 32
	(bars)	$n_{CR_{1}}$	10	3 to 12
	(m)	$H_{CR_{1}}$	76 to 226	1.5 to 0.375 ·L
	(m)	$V_{CR_{1}}$	76	1 to 2.5
Corner reinforcement $CR_{2}$	(mm)	$\phi_{CR_{2}}$	6	10 to 32
	(bars)	$n_{CR_{2}}$	10	3 to 12
	(m)	$H_{CR_{2}}$	101 to 251	1 to 0.375 ·L
	(m)	$V_{CR_{2}}$	26	1 to 1.5
Shear reinforcement $SR_{1}$	(mm)	$\phi_{SR_{1}}$	7	8 to 32
	(m)	$s_{SR_{1}}$	7	0.10 to 0.40
	(m)	$LD_{SR_{1}}$	76 to 226	1.5 to 0.375 ·L
Shear reinforcement $SR_{2}$	(mm)	$\phi_{SR_{2}}$	7	8 to 32
	(m)	$s_{SR_{2}}$	7	0.10 to 0.40
	(m)	$LD_{SR_{2}}$	101 to 251	1 to 0.375 ·L

**Table 3:** Main parameters considered in the RMHF optimization.

Geometrical Parameters		
Free height (m)	*H*	5
Horizontal span (m)	*L*	8 to 16
Hinge height (m)	HH	(3/5) · *H*
Earth cover (m)	HE	1 to 5
**Loading parameters**		
Earth specific weight (kN/m${}^{3}$)	$\gamma_{E}$	20
Reinforced concrete specific weight (kN/m${}^{3}$)	$\gamma_{C}$	25
Earth internal friction angle (${}^{\circ }$)	IF	30
Active earth pressure coefficient	$K_{A}$	0.33
Resting earth pressure coefficient	$K_{R}$	0.50
Heavy traffic vehicle load (kN/m${}^{3}$)	TL	150
Heavy traffic vehicle load lenght (m)	TLL	1.20
Uniform overload (kN/m${}^{3}$)	UO	10
Ballast coefficient (MN/m${}^{3}$)	$B_{E}$	10
**Economic and sustainability parameters**		
Unit costs (EUR)	$c_{i}$	Table 1
Unit CO_2_ emissions (CO_2_ kg)	$ae_{i}$	Table 1
Unit embodied energy (kWh)	$ee_{i}$	Table 1
**Exposure related parameters**		
Exposure class	XC2	
**Legislative related parameters**		
Standard regulations	CEN [30,31]/MFOM [32]	
Code considerations	MFOM [33]	

**Table 4:** Metaheuristic parameter bounds considered in the DoE.

SAMO	P1	P2	P3	P4	P5
Parameter	MCL	SD	VN	CC	SC
Lower bound (−)	1000	0.1	1	0.8	1
Upper bound (+)	5000	0.3	5	0.9	5
**TAMO**	**P1**	**P2**	**P3**	**P4**	**P5**
Parameter	CL	SD	VN	RC	SC
Lower bound (−)	1000	0.1	1	0.8	1
Upper bound (+)	5000	0.3	5	0.9	5
**OBAMO**	**P1**	**P2**	**P3**	**P4**	**P5**
Parameter	TC	SD	VN	IC	DC
Lower bound (−)	10,000	0.1	1	1	1
Upper bound (+)	50,000	0.3	5	5	5

**Table 5:** DoE mean minimum cost results and associated computational cost for each configuration.

					SAMO			TAMO			OBAMO		
**P1**	**P2**	**P3**	**P4**	**P5**	**Cost (EUR)**	**Iter.**	**% Min.**	**Cost (EUR)**	**Iter.**	**% Min.**	**Cost (EUR)**	**Iter.**	**% Min.**
−	−	−	−	+	3722.99	11,472	0.0067	4484.78	7790	0.2127	4266.87	5621	0.1538
+	−	−	−	−	3700.97	56,759	0.0008	4206.81	28,405	0.1376	3935.18	15,901	0.0641
−	+	−	−	−	3809.90	10,551	0.0302	4005.26	6823	0.0831	3939.44	6688	0.0653
+	+	−	−	+	3723.28	44,999	0.0068	3933.48	19,124	0.0636	4108.21	30,886	0.1109
−	−	+	−	−	3893.24	13,428	0.0528	3844.05	14,008	0.0395	4131.20	6607	0.1171
+	−	+	−	+	3764.81	75,442	0.0180	3764.61	64,237	0.0180	4420.60	31,150	0.1954
−	+	+	−	+	3854.21	14,101	0.0422	3917.74	12,470	0.0594	4470.99	3043	0.2090
+	+	+	−	−	3787.60	76,644	0.0242	3764.19	62,072	0.0179	3978.78	23,037	0.0759
−	−	−	+	−	3759.89	32,726	0.0167	3900.58	20,817	0.0548	4233.84	5175	0.1449
+	−	−	+	+	3735.80	137,994	0.0102	3990.50	61,719	0.0791	3808.35	30,193	0.0298
−	+	−	+	+	3745.25	38,517	0.0127	3953.74	22,026	0.0691	3878.68	7326	0.0488
+	+	−	+	−	3717.33	135,874	0.0052	4376.86	41,030	0.1835	4103.01	28,609	0.1095
−	−	+	+	+	3810.35	53,194	0.0304	3759.25	53,703	0.0165	4135.06	8395	0.1182
+	−	+	+	−	3740.23	254,796	0.0114	3711.42	227,138	0.0036	3788.71	30,451	0.0245
−	+	+	+	−	3808.14	52,211	0.0298	3755.12	52,050	0.0154	4188.70	4486	0.1327
+	+	+	+	+	3730.33	266,381	0.0087	3698.10	258,351	0.0000	3838.12	43,960	0.0379

## Data Availability

All the data used in the research can be found in the article.

## Abbreviations

The following abbreviations are used in this manuscript:

SA	Simulated Annealing
TA	Threshold Accepting
OBA	Old Bachelor’s Acceptance
MO	Mutation Operator
GA	Genetic Algorithm
RMHF	Road Modular Hinged Frame
CPRF	Cast in Place Road Frame
DoE	Design of Experiments
SAMO	Simulated Annealing with Mutation Operator
TAMO	Threshold Accepting with Mutation Operator
OBMO	Old Bachelor’s Acceptance with Mutation Operator
ULS	Ultimate Limit State
SLS	Service Limit State
MCL	Markov Chain Length
CC	Cooling Coefficient
SC	Stopping Criterion
VN	Variable Number
SD	Standard Deviation
CL	Chain Length
RC	Reduction Coefficient
TC	Termination Criterion
IC	Increase Coefficient
DC	Decrease Coefficient
